# Supplementary material for: The bacterial toxin colibactin triggers prophage induction
Source: Nature. 2022 Feb 23;603(7900):315–20. doi: 10.1038/s41586-022-04444-3 (PMC8907063; doi:10.1038/s41586-022-04444-3)
Supplement: Supplementary file 1 — This file contains additional discussion pertinent to study findings; the caption for Supplementary Table 1; Supplementary Tables 2–4; and the Supplementary References. [file 41586_2022_4444_MOESM1_ESM.pdf]

nature portfolio

https://doi.org/10.1038/s41586-022-04444-3

Supplementary information

The bacterial toxin colibactin triggers prophage induction

In the format provided by the authors and unedited

Supplementary information for

The bacterial toxin colibactin triggers prophage induction

Justin E. Silpe, Joel W. H. Wong et al.

Supplementary Discussion | Additional discussion pertinent to study findings.

I. Prophage induction by a native colibactin producer.

Many studies of colibactin employ strains that heterologously express the *pks* cluster on a BAC.[1] This strategy has proven useful for elucidating colibactin's structure and biosynthesis, but it may fail to capture regulatory features of native colibactin-producing strains. To address this uncertainty, we tested a native colibactin producer, *E. coli* NC101, a murine adherent-invasive strain isolated from mouse models of CRC carcinogenesis.[2,3] As shown in Extended Data Figure 2b and c, WT NC101 activated the P_R-*lux* reporter and increased phage titers by approximately 2 orders of magnitude compared to the NC101 Δ*clbP* mutant. While the number of plaques produced was generally lower from the NC101 co-cultures than the heterologously expressed BAC-*pks*, the result demonstrates that a native colibactin-producer induces the SOS response and activates prophages in neighboring cells in a *pks*-dependent manner, and in comparable magnitude compared to the BAC-*pks*-containing laboratory *E. coli*. Moreover, our reporter data showing that P_R-*lux* activity is completely abolished when exposed to the NC101 Δ*clbP* mutant, suggests that, under the conditions tested, colibactin production is likely the only mechanism by which NC101 activates the SOS response in neighboring *E. coli* (Extended Data Figure 2b).

II. The use and specificity of extracellular DNA to interfere with colibactin-dependent prophage induction

As noted in the main text and displayed in Extended Data Figure 2d-h, we used extracellular DNA (eDNA) as a means of exogenously manipulating the amount of colibactin delivered to target organisms. The rationale for this approach was that, in mammalian cell systems, addition of eDNA

has been shown to attenuate the genotoxicity of *pks*$^+$ *E. coli*, presumably by titrating away the reactive colibactin through alkylation of the exogenous DNA (we observed a similar effect in our setup using herring sperm DNA, Extended Data Figure 2e and f).[4] To investigate the importance of the AT-GC richness of the DNA used in this assay, we designed short oligonucleotides with variable AT:GC ratios relative to herring sperm DNA (58% AT). As referenced in the main text (Extended Data Figure 2g and h), AT-rich DNA (75% AT) but not GC-rich DNA (29% AT) attenuated prophage-induction, both in terms of reporter output and plaques produced. Notably, the pattern we observe for AT- versus GC-rich DNA in our assay is consistent with recent reports of colibactin-induced DNA damage and its concomitant mutational signatures occurring predominantly at AT-rich motifs.[5,6]

III. Bioinformatic prediction of prophages in clbS+ non colibactin producers

We reasoned that protection against colibactin-mediated DNA damage could provide a mechanism by which these diverse bacteria can avoid induction of native prophages they might carry. Thus, we searched for resident prophages in the available genomic data of the 12 bacteria from which the ClbS panel was assembled (Extended Data Figure 4a). Using a prophage-prediction algorithm (PHASTER),[7] we found a total of 94 prophage regions in the 12 genomes (Extended Data Figure 4b and c), 9 of which were further classified as containing at least one intact prophage (score >90, Extended Data Figure 4c). Like their bacterial hosts, these putative prophages are largely uncharacterized, however, we hypothesize based on domain analysis of the predicted prophage repressors that a subset of these prophages are responsive to DNA damage in a manner similar to phage lambda (Extended Data Figure 4d).

IV. Phage-independent differences in sensitivity to colibactin and mitomycin C by S. aureus and E. coli.

In our investigations, we observed several notable differences in phage-dependent versus phage-independent responses to colibactin between *S. aureus* and *E. coli.* In particular, the two prophage-

carrying *S. aureus* strains used in this study underwent a four order of magnitude drop in colony forming units (CFU) when cultured with pks^+ versus pks^- *E. coli*, as compared to a two order of magnitude drop observed for phage-free *S. aureus* (Supplementary Discussion Figure 1a). The reduction of CFUs even in phage-free strains of *S. aureus* contrasts with our earlier finding on the lack of growth inhibition in phage-free *E. coli* (Figure 1b and Extended Data Figure 1a and b), indicating that bacteria can differ in their response to colibactin.[8] While a complete understanding of this difference remains to be resolved, we suspect it is a feature of DNA-damaging agents more generally as phage-free *S. aureus* was significantly more sensitive to MMC than phage-free *E. coli* (Supplementary Discussion Figure 1b).

Supplemental Discussion Figure 1

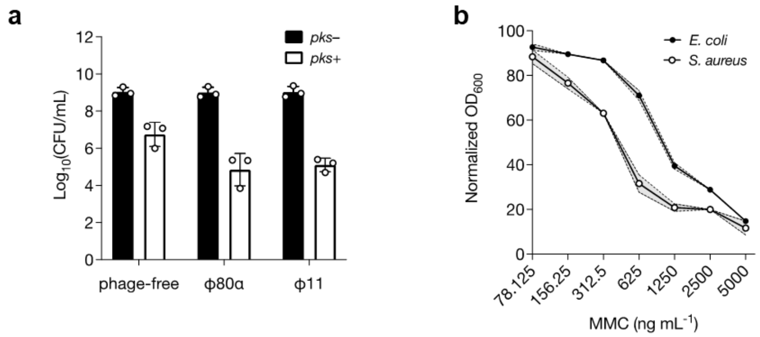

Supplementary Discussion Figure 1 | *S. aureus* prophages are highly sensitive to colibactin, and *S. aureus* in general is likely more susceptible to other DNA-damaging agents than phage-free *E. coli*.
a Colony forming units (CFU) of lysogenic and non-lysogenic (phage-free) *S. aureus* RN450 after being co-cultured with pks^+ and pks^- *E. coli.* **b** Growth of phage-free strain of *S. aureus* and *E. coli* in the presence of varying doses of MMC. Normalized OD_{600} was calculated as the OD_{600} at a given concentration relative to the OD_{600} of the same strain to which no MMC was added (defined as 100). Data for both panels represented as mean ± SD with n = 3 biological replicates.

V. Broader implications of colibactin production on prophage induction in complex communities:

In this study, we investigated the interaction between colibactin-producing and -susceptible bacteria. We show that exposure to colibactin activates lytic development in prophage-carrying bacteria and that this effect occurs via the canonical SOS response. Notably, we observe that colibactin is an effective inducer of prophages across diverse phage-host systems and functions in complex communities. We also find many examples of non-colibactin-producing bacteria that possess colibactin resistance genes, indicating past exposure to this genotoxin. We propose that

the occurrence of colibactin resistance in these non-producing organisms serves as a unique defense mechanism to avoid prophage-mediated cell lysis and to prevent phage outbreaks caused by colibactin producers within a given community. Here, we outline several potentially important roles for colibactin and other bacterial natural products in modulating the activity of phages in microbiomes.

The virulence of certain pathogenic bacteria depends on their lysogenic state, and it is thus interesting to consider how colibactin might operate in these situations either positively, by helping to eliminate the pathogen, or negatively, by activating the expression of prophage-controlled virulence factors the pathogen harbors. For example, in the case of Stx-producing bacteria, the use of quinolone antibiotics is specifically contraindicated because it increases the induction of the SOS-responsive *stx* prophage and concomitant production of the encoded Stx toxin,[9] an effect we also observe with colibactin.

It is also interesting to compare how phage-mediated lysis compares to more direct means of cell-killing and competition (e.g. production of bacteria-encoded toxins). For example, if there were members of the bacterial community susceptible to phages induced by colibactin, production could indirectly stimulate a phage outbreak. By inducing phage outbreaks in microbial communities, colibactin could impact bacteria beyond those which are directly exposed to the metabolite itself. Furthermore, phage infections that lead to lysogeny could have lasting consequences on the infected population as the prophage will be inherited by future generations. We also envision scenarios where prophage induction could be counterproductive for colibactin producers. For example, in a community of closely related species or strains of the same species, phage induction may create phage particles that can go on to infect the colibactin producer, if susceptible. Nevertheless, in most microbial habitats where phages are thought to significantly outnumber the bacterial population,[10,11] it is likely the colibactin producer itself is also a lysogen and hence immune from superinfection. Such a scenario has been examined in a prophage-containing clinical isolate of *Enterococcus faecalis*, where the presence of the resident prophage enhances this isolate's colonization over other closely related phage-susceptible strains both in vitro and in vivo.[12]

Supplementary Table 1| tBLASTn result of top 230 *clbS*-like genes encoding proteins matching *E. coli* ClbS, WP_000290498 (tab 1); BLASTp result of top 5,000 ClbS-like proteins using *E. coli* ClbS, WP_000290498 as query (tab 2, in separate excel).

Supplementary Table 2| Strains used in this study.

Strain	Genotype	Reference
E. coli DH10β	*F– mcrA Δ(mrr-hsdRMS-mcrBC) φ80lacZΔM15 ΔlacX74 recA1 araD139 Δ(ara-leu)7697 galU galK λ– rpsL(*Str^R*) endA1 nupG*	NEB
E. coli BW25113	*lacIq, rrnBT14, ΔlacZWJ16, hsdR514,ΔaraBADAH33, ΔrhaBADLD78*	Baba et al. 2006 [13]
E. coli BW25113 λ	BW25113 lysogenic for λ	This study
E. coli BW25113 *lamB::kan*	*lacIq, rrnBT14, ΔlacZWJ16, hsdR514,ΔaraBADAH33, ΔrhaBADLD7 ΔlamB732::kan*	Baba et al. 2006 [13] (JW3996-1)
E. coli MG1655 KIlac	MG1655 with Lac Operon removed from native locus and re-integrated at the Tn7 locus	Eames et al. 2012 [14] (Addgene: #52696)
E. coli MG1655 (delta-Z)	KIlac with *lacZ* deletion	Eames et al. 2012 [14] (Addgene: #52706)
E. coli NC101	Murine isolate of *E. coli* (causes colitis in gnotobiotic IL-10−/− mice)	Tomkovich et al. 2017 [15]
E. coli NC101 Δ*clbP*	Δ*clbP* mutant of *E. coli* NC101	Tomkovich et al. 2017 [15]
S. Typhimurium D23580 ΔΦ (JH3949)	D23580, cured of 5 native prophages	Owen et al. 2017 [16]
S. Typhimurium D23580 ΔΦ [P22] (SSO128)	D23580 ΔΦ lysogenic for P22	Owen et al. 2021 [17]
S. Typhimurium polylysogen (SVO2)	D23580 BTP1, Gifsy-1 polylysogen	Owen et al. 2017 [16]
S. Typhimurium ΔBTP1 (BL1)	D23580 ΔBTP1	Owen et al. 2017 [16]
S. Typhimurium ΔGifsy-1 (BL2)	D23580 ΔΦ Δ*waaG*::*aph*	This study
C. rodentium (Φstx_{2dact})	DBS770	Mallick et al. 2012 [18]

S. aureus RN450	NCTC8325 cured of Φ11, Φ12, Φ13	Novick 1967 [19]
S. aureus RN10359	RN450 lysogenic for Φ80a	Úbeda et al. 2007 [20]
S. aureus RN451	RN450 lysogenic for Φ11	Novick 1967 [19]
Enterococcus faecium E1007	Isolated from feces of a healthy individual	Lebreton et al. 2017 [21]
Escherichia albertii 07-3866	*Escherichia albertii* isolated from human in the U.S.	Lindsey et al. 2019 [22]

Supplementary Table 3 | Plasmids used in this study.

Plasmid	Relevant fragment	Resistance	Reference
JSS-2347	empty BAC (pBeloBAC11)	Cm	NEB
JSS-2348	BAC-*pks*	Cm	Gift of Bonnet Lab
JSS-2367	BAC-*pks* Δ*clbP*	Kan Cm	Gift of Bonnet Lab
JSS-2435	empty BAC2 (cos sites removed)	Cm	This study
JSS-2403	P_R-*lux*	Kan	This study
JSS-2587	pBR322-empty (*ampR*)	Amp	This study
JSS-2436	pEVS143-empty (*kanR*)	Kan	This study
JSS-2442	pEVS143-empty (*kanR-cmR*)	Kan Cm	This study
JSS-2470	pTrc-*clbS*	Amp	This study
JSS-2696	pTrc-*clbS*	Kan	This study
JSS-2482	pTrc-Δ*clbS* (pTrc-*clbS* with *clbS* deleted)	Amp	This study
JSS-2522	pTrc-$clbS_{Dickeya}$ (*Dickeya dadantii*)	Amp	This study
JSS-2523	pTrc-$clbS_{Mixta}$ (*Mixta theicola*)	Amp	This study
JSS-2528	pTrc-$clbS_{Ecoli69}$ (*Escherichia coli 69,* plasmid)	Amp	This study

JSS-2529	pTrc-*clbS*$_{Snod}$ *(Snodgrassella alvi wkB2)*	Amp	This study
JSS-2530	pTrc-*clbS*$_{Gibsiella}$ (*Gibbsiella quercinecans* DSM 25889)	Amp	This study
JSS-2531	pTrc-*clbS*$_{Samsonia}$ (*Samsonia erythrinae* DSM 16730)	Amp	This study
JSS-2532	pTrc-*clbS*$_{Frischella}$ (*Frischella perrara*)	Amp	This study
JSS-2555	pTrc-*clbS*$_{Bifido}$ (*Bifidobacterium longum subsp. infantis*)	Amp	This study
JSS-2556	pTrc-*clbS*$_{Ealbertii}$ (*Escherichia albertii* CB9786)	Amp	This study
JSS-2557	pTrc-*clbS*$_{Metako}$ (*Metakosakonia sp.* MRY16-398)	Amp	This study
JSS-2576	P_{Metako}-*lux*	Kan	This study

Supplementary Table 4 | Gene blocks and oligonucleotides used in this study.

ID	Sequence (5-3')
JSgblock-0133 (*Dickeya dadantii*)	ATATTAATGTATCGATTAAATAAGGAGGAATAAACCATGGGCGTGCCACAAACAAAATCCGAACTACT<br>TTTCGCTATTGATAAAAATTTTACGAAATTAATCGGTTACCTCAATGCGATTCCGCCAGAGCTCACTG<br>CGGATAAATCGCTGGATGGCCATGCAAAAGGCACGGAGATGAGTGTTTGTGAGCTTGTTTCGTATTT<br>GCTGGGATGGAACACTCTGGTTGTAAAATGGATTAGCGGTGACGCAACAGGGCAACCTGTTGATTT<br>CCCGGAAACCGGTTACAAGTGGAATCAGCTCGGGCTTCTGGCGCAGAAATTTTACTTTGATTACAAA<br>GAATTAAATTACCAGACGTTAATCAATAACCTTCAGTCGGTAAAAGACGAAATAGTGAGGCTGATTGA<br>TGAGCGCACTGATGAAGATCTGTATGGCAAGCCATGGTATGGCAAATGGACAATGGGACGTATGAT<br>CTCATTGAACACGTCATCACCCTATGCCAATGCTAATGGAAGATTAAGGCAGTGGGCAAAGAACAAT<br>CACCTGCGTTTAAAATGAGTTTTGGCGGATGAGAGAAGATTTTCAGCCTGATAC
JSgblock-0134 (*Frischella perrara*)	ATATTAATGTATCGATTAAATAAGGAGGAATAAACCATGGCAGTACCTGAATCTAAAACTGAGTTAAT<br>AGAAGCGATTAATAAAAATTATGCTTCGTTAGTCAAAAAGCTATCAGCTGTACCAGCAGAAAAGGCTT<br>TCCAACCATTAATGGAGGGGCATGCCAAAGGCACGACTATGAGTGCAGCACAACTTGTTTCTTATTT<br>AATAGGTTGGGGCGAATTAGTCTTATCTTGGCATGCACAAGAAGCACATGGGCATAGCATCGTTTTT<br>CCTGAAGTGGGCTTTAAATGGAATGAATTAGGAAAACTAGCACAGAAATTTTATCAAGACTATCAAAA<br>CATCACTGATTACAAGGAGCTATTACAACGGTTAGAAAAAAATAAAAATGATCTTATTAAATTGATTGA<br>AGGTTTTACGAATAATGATTTATATGGTTCGAGTTGGTGTGAAAAATACACTCGAGGACGAATGATTC<br>AGCTAAATACGTCATCACCGTATAGAAATGCAAGTGGTCGCATCGCGACATTCCTCAAAACTATCGG<br>CAATTAAGTTTTGGCGGATGAGAGAAGATTTTCAGCCTGATAC
JSgblock-0135 (*Mixta theicola*)	ATATTAATGTATCGATTAAATAAGGAGGAATAAACCATGGCGGTACCGGGATCAAAAGCTGAATTAAT<br>TAAAGCGATCAATAGTAATTTCGCGTTGCTGAATAAAAAGCTGGAGGCTATTGCGCCAGCGCTTGCC<br>TTTGAGCCGCTTATGGAAGGGCATGCCAAAGGGAGCACGATGAGCGTGGCGCAGCTGGTCTCCTG<br>GCTGATTGGCTGGGGCGAATTGGTGTTGCACTGGCATAACCAAGAGGCGAAAGGGGAAACCATCGT<br>GTTTCCTGCAGAGGGCTATAAATGGAATGAGCTGGGACAGCTGGCGCAGAAATTCTACCGTGACTA<br>TGAAGACATCAACAATTATGAAACCTTGTTGGCGCGGTTAAGGGAGAACAAACAGCGGCTCTTGATG<br>CTGACTGAGCAATTTAGCAACGATGAACTTTACGGTACGCCCTGGTATGGCAAATGGACGCGAGGA<br>CGAATGATTCAATTTAATACCGCCTCGCCCTATAGAAATGCCGCAGGACGATTAAACAAATTGAAAAA<br>ACAAACTGCTGCGTAAGTTTTGGCGGATGAGAGAAGATTTTCAGCCTGATAC
JSgblock-0136 (*Escherichia coli* 69, plasmid)	ATATTAATGTATCGATTAAATAAGGAGGAATAAACCATGAGTGTGCCGCAAACAAAAGCTGAACTGCT<br>TTTAGCTATTGATAAAAATTTTAGTAAATTAATTAGTTACCTCAACACAATCCCACCAGAAATTACTTCA<br>GATAAATCAATGGACGGACACGCCAAAGGAACGGAGATGAGTGTTCGTGATCTCGTTTCGTATCTGC<br>TTGGATGGAATGCTCTTGTTGTAAAGTGGATCGCTTCTGATGCTAAAGGTCTGCCTGTCGATTTTCC<br>GGAAACTGGCTATAAATGGAATCAGCTTGGCCTTCTTGCTCAAAAATTTTACTCAGATTACAGTGAGT<br>TAAGTTATGAGTTGTTAGTAGCTGAACTTCAGACTGTAAAAAATGAGATTGTGAACCTTATTAATGATC<br>GTACCGATGATATTTTGTATGGAAGACCATGGTACACAAAATGGACGATGGGGAGAATGATCTCATT<br>TAACACATCTTCGCCTTACGCCAACGCTAATGGAAGATTAAGAAAGTGGGCAAAAAATAATAATATCA<br>GTTTAAAGTAAGTTTTGGCGGATGAGAGAAGATTTTCAGCCTGATAC
JSgblock-0137 (*Snodgrassella alvi* wkB2)	ATATTAATGTATCGATTAAATAAGGAGGAATAAACCATGGCAATACCAGAATCAAAACAGGAACTAAT<br>TGAAGCAATTAATAAAAACTATACACTACTGACCAAAAAGCTGGCTGCTGTACCTGAACAGAAAGCCT<br>ATCTGCCTCTGATGGAAGGCCATGCTAAAGGTACAATGATGAGTGCTGCACAGCTTGTGTCTTATTT<br>AATTGGCTGGGGAGAACTGGTGCTGTCATGGCATAAACAGGAACAGTCAGGGCAGAAAATTGCCTT<br>TCCGGAAGCAGGATATAAATGGAATCAACTGGGATTACTGGCACAGAAATTTTATCGTGATTATCAG<br>GACATTACCGATTTCAAACAGTTACTCTCGCTTCTGGAGACCAACAAAAAGAATCTGATTTCACTTAT<br>TGATAGCTTTAGTAATGAAGAGCTGTATGGCTCACCCTGGCATGAAAAATACACACGCGGACGCATG<br>ATTCAGTTTAATACCTCCTCCCCTTATAAAAATGCCACAGGACGATTGAATAAATTGTTGAAACAGAT<br>CAAAGAATAAGTTTTGGCGGATGAGAGAAGATTTTCAGCCTGATAC
JSgblock-0138 (*Gibbsiella quercinecans* DSM 25889)	ATATTAATGTATCGATTAAATAAGGAGGAATAAACCATGGCCGTGCCGGAATCAAAAGGCGCATTGA<br>TCAAAGCGGTGAACAGCAATTTCGCGCTGCTGATGAAAAAACTGGACGACATACCGGCAGAAAAGG<br>CGTTTGAGCCGCTGATGCCGGGGCATGCCAAGGGCAGCGTAATGAGCGTTGCCCAACTGGTGGCC<br>TATCTGATTGGCTGGGGGGAACTGGTTTTGTCCTGGCATGCGAAAGAAAGACAGGGCCTTGAAATC<br>GCCTTCCCCGAAGACGGTTTTAAGTGGAACCAATTGGGGTTGCTGGCGCAAAAGTTTTACCGTGATT<br>ATGCGCAGATTACGGACTACGGCGCCCTGCTGATGCGGCTTGCCGATAATAAACGGCAGATTATCG<br>CGTTGATTGACGGCTTTAGCGATGAGGCGCTCTACGGCCAGCCGTGGTACGGCAAATGGACGCGT<br>GGCCGGATGATTCAGTTCAACACGGCGTCACCGTATAAGAACGCCTGCGGCAGGCTAAACGCATTG<br>CGAAAAGTAGGCTGTGTCCCGTAAGTTTTGGCGGATGAGAGAAGATTTTCAGCCTGATAC
JSgblock-0139 (*Samsonia erythrinae* DSM 16730)	ATATTAATGTATCGATTAAATAAGGAGGAATAAACCATGGCGGTGCCAGAATCAAAAGATGAACTCAT<br>TAAAGCGATCAATAGTCACTTTGCGTTATTGCAGAAAAAGCTGGGGGCAGTCCTGCCTGAGCAGTCC<br>TTTGAACCGACAATGGAAGGGCATGCAAAAGGGAGCGTGATGAGCGTGGCCCAATTGGTGTCTTAT<br>CTTATTGGGTGGGGAGAATTGGTCTTGCACTGGCACGAACAAGAAGCGAAAGGGGAAACGATAAAC<br>TTTCCTGAAGAGGGCTATAAATGGAATGAATTGGGGCGCCTGGCGCAGAAGTTTTATCGCGACTATG<br>AAGACATAAAGGATTATGAAATCTTATTGTTGCGATTAAAGGAAAACAAACAGCAGCTTTTGACGCTG<br>ATTGAGCGCTTTAGTAACGAGGAACTTTACGGTAGTCCTTGGTATGGCAAATGGACGCGGGGGCGA<br>ATGATTCAATTTAATACCTCCTCGCCTTATAAAAATGCTTCAGGGCGATTAAATAAATTGAAAAAACAG<br>GCTCCGATATGAGTTTTGGCGGATGAGAGAAGATTTTCAGCCTGATAC
JSgblock-0140 (*Bifidobacterium longum subsp. infantis*)	ATATTAATGTATCGATTAAATAAGGAGGAATAAACC.ATGGCAGCCGGGAGGTGCATAGTGAGAACAT<br>ACGAGAACGCGCAGGAGCTCAAAAAGGAGATCGGCGCGGCGTTTCGAAAATACATTGCGGAGTTCG<br>ACGACATTCCGGAAGCCCTGAAAGACAAGCGCATCGACGAGGTCGAGAGGACCCCGGCGGAAAAC<br>CTCGCCTATCAGGTTGGTTGGACCACCCTGCTGCTGCAATGGGAGGATCGCGAGCGGAAGGGCCT<br>TCCGGTGCGAACGCCGTCGGACGAGTTCAAGTGGAACCAGCTCGGAAAGCTGTACCAGTGGTTCAC<br>CGACACCTACGCCCACCTTTCGCTGCGGGAGCTGAAGGGCATGTTGACCGACAACGTCGACGCCAT<br>ATACGCCATGATCGATGCGATGAGCGAGGACGAGCTGTTCAAGCCGCATATGAGGAAGTGGGCCG<br>ACGACGCCACCAAAACGGCGGTATGGGAAGTGTACAGGTTCATTCATGTGAACACCGTCGCCCCGT<br>TCGGATCGTTCAGGACGAAGATCCGCAAGTGGAAGCGGATGGCCCTATAG.GTTTTGGCGGATGAG<br>AGAAGATTTTCAGCCTGATAC

JSgblock-0142 (*Escherichia albertii* CB9786)	ATATTAATGTATCGATTAAATAAGGAGGAATAAACC.ATGTCTGTACCAGAAAGCAAAGCTGCACTGTT<br>GCTGGAAATGGAAAAATCATGGAATGGACTTACAAAAAAGTTATCTCGCATTCCAGAAGAAAAAGCTT<br>TTCTCATCACAATGGAAGGACATGCCAGCGGCACTGTGATGAGTGCGGCTAACCTGGTAAGCTATTT<br>AATAGGTTGGGGAGAGCAGGTTCTTACCTGGCACAGACAAGAAGAAGCAGGAATCCCCATTGATTTT<br>CCGGCTAAAGACCATAAGTGGAATGAACTTGGGAAACTAGCGCAGACATTTTACGCTAATTATTCCC<br>ATGTCACATCATGGTCCCAATTGTGCGAAATGTTAGAAACCAATCATCAGCAACTCAAATCGCTAGTG<br>GAACGCTACAGCGATAACGAGTTATACCATCATCCCTGGTATGGCAAATGGACGCGTGGTCGAATG<br>ATCCAATTTAATACCGTATCACCCTATAGAAATGCAAGCACACGTCTGAACGCCCTACTTAAACAATT<br>CTGAGTTTTGGCGGATGA.GAGAAGATTTTCAGCCTGATAC
JSgblock-0143 (*Metakosakonia* sp. MRY16-398 / *Kluyvera intestini* strain GT-16)	ATATTAATGTATCGATTAAATAAGGAGGAATAAACCATGGCCGTGCCAGAAAGCAAAACTGCCTTACT<br>GGAAGCAATGGAAAAGTCGGCAAGCGCCCTGCGCAAAAAACTCATCCGCATTCCGGCGGACATCGC<br>TTATCAGCAATGCCTGGAAGGGCACGTGGCTGGTAGCCGCATGAGTGTTGCTAATCTTGTCAGCTAT<br>CTGATTGGCTGGGGAGAGCAAGTTCTCTTCTGGCACCAACAGGAAGCCGCAGGCGAAGAAATAGAC<br>TTCCCGGCAAAAGGCTTTAAATGGAATGAACTGGGGAAGCTTGCGCAAAAATACTACGCTGATTACC<br>AGCATATTACGTCATGGCCAACGCTGCTTGCCATGCTGGAAGAGAATCAACAGCAACTGATATCACT<br>GGTGAATGGCTTTAGTGACGATGAGTTGTACCACCAGCTCTGGTACGGAAAATGGACGCGAGGACG<br>TATGATTCAATTCAACAGCGCCTCACCGTATAAGAATGCCAGCGCCAGGCTGAACAGTCTGTTAAAA<br>ACGCTCGCCGAAGCCTGACGCTCGCCGAAGCCTGAGTTTTGGCGGATGAGAGAAGATTTTCAGCCT<br>GATAC
JSgblock-0151 (*Metakosakonia* sp. MRY16-398_2 phage)	CGGGTACCGCTCGAGTTAATTAAGGCATAAAAAAACCAGCCGAAGCTGGTTTTTTATATGTATGAGT<br>CTCACCCCAAACGGTTGTATGACATTTGCCATCTACCGACAACTAAGCCCTGAATGTGAAACTCGCT<br>TTGATCAGTTTCAGATACAAACCATTTTTCGTAAGTTGAATTATCGCTGATGACTACCAGCTTGTCCTT<br>AAGCAGCTGTAAGCGTTTGATATGAAAGTTGTCACCATAAACGAACGCATAAATCCCATCACCTATCA<br>ACCGGTTTACCGTAACGTCTATAACGACAAGATCCCCAGGGAAAATAGTCCCGAGCATGCTATCGCC<br>GACAACCGTGCAGATTTTCAGGGAGGTAGGGCTACGCCCACCAAACATCCTTTTAGCTTCCTCTGGA<br>TCAAGTTCAATCGATTTAACTATTTCAGGATAGTCCATGTTCATCCTGCCACTTCCGCAACTGAACTC<br>AGTGTCCAGCACCTCAATTCGGTATTGTTGCTGATTTTTTGTCAGTGGATTTATGAAAAAGTTAGGCT<br>TGGTACCAGATGCATCAACATCTGCTCTCTTTGAATCCATCCAGCCATGGGGCAGGCCCATACTTGA<br>CTCAATTCTCCTAGCTATTATGTCACCAATGTTACGAGAGGCGTTAGAGCCAGATAACTGACTCAGTT<br>GAGAAGGGGGTATACCAATTTTTTCAGCAATTTGTGCCTTGGTATAACCTTCCACGATGTACTCACCA<br>ATCAACCTGTTTAGGTTCTGACGTCGAATGTTTTTTATGTCCATGACCAAATACTCCCACTTTTTAGCA<br>ATATGATAAATACCCAATTTGATAAATTTATCTTGCCTCATAGTTATCGTAAAGATAAACTTTACCAAAA<br>TGATAAATTGAGGTGTGGGTATGACTAAAAAAATTTCATTCATTATTAACGGCCAG
JSO-1758: pTrc backbone	GTTTTGGCGGATGAGAGAAG
JSO-1759: pTrc backbone	GGTTTATTCCTCCTTATTTAATCGATACATTAA
JSO-1756: *clbS* (into pTrc)	ATGGCTGTTCCATCATCAAAAGAAGAG
JSO-1757: *clbS* (into pTrc)	CTATTCTGCAAGACATTTCTGCAGTTTATTTAACC
JSO-1766: *clbS* deletion from pTrc-*clbS* (construction of pTrc-Δ*clbS*)	TTCTTTTGATGATGGAACAGCCAT
JSO-1767: *clbS* deletion from pTrc-*clbS* (construction of pTrc-Δ*clbS*)	CTGCAGAAATGTCTTGCAGAATAG
JSO-1727: P_R-*lux* insert	TGAATGAAATTTTTTTAGTCATACAACCTCCTTAGTACATGCA
JSO-1730: P_R-*lux* insert	GGTACCGCTCGAGTTAATTAAGATCAGCCAAACGTCTCTTC
JSO-1691: P_R-*lux*, P_{metako}-*lux* and pEVS143-empty (kanR)	TTAATTAACTCGAGCGGTACCCG
JSO-1692: P_R-*lux* and P_{metako}-*lux*	ATGACTAAAAAAATTTCATTCATTATTAACGGCCAG
JSO-1731: pBR322-empty (*ampR*)	TTAATTAACTCGAGCGGTACCTCTTAC
JSO-1743: pBR322-empty (*ampR*) and pEVS143-empty (*kanR*, with JSO-1691)	GGATCCGGTGATTGATTGAGC
JSO-1741: empty BAC2 (cos sites removed)	GTATTTTGTCCACATAACCGTGCG
JSO-1742: empty BAC2 (cos sites removed) and cmR (with JSO-1745) for pEVS143-empty (*kanR-cmR*, ligated with primer product used for pEVS143-empty *kanR*)	GAGTGAGCTAACTCACATTAATTGCG
JSO-1998: pTrc-clbS *ampR* to *kanR*	GCCCTCTCACTTCCCTGTTAAG
JSO-1999: pTrc-clbS *ampR* to *kanR*	TCAAGGTGTACTGCCTTCCAG
JSO-2000: pTrc-clbS *ampR* to *kanR*	GCATTGGTAACTGTCAGACCAAGT
JSO-2001: pTrc-clbS *ampR* to *kanR*	GGGTTATTGTCTCATGAGCGGATAC
JSO-1973: lambda red *clbS* recombineering from *E. albertii*	GGACCATGGCTAATTCCCATCAAATATATTGTCAGAATTGTTTAAGTAGGGCGTT
JSO-1972: lambda red *clbS* recombineering from *E. albertii*	TCACTATGCGCTGTAAAACGC

JSO-1971: lambda red *clbS* recombineering from *E. albertii*	GTGATGAAAGGTCTATTATCATCGGC
JSO-1970: lambda red *clbS* recombineering from *E. albertii*	GAAGCAGCTCCAGCCTACACCTATTTATTCGCTGATTTTTCTGCTCGT
JSO-1969: lambda red *clbS* recombineering from *E. albertii*	GTTGATGGAAATTGTTATGCCTCAGA
JSO-1968: lambda red *clbS* recombineering from *E. albertii*	AGCATGAACCCGATGTTACC
JSO-1967: pKD3 *cmR* amplification	GAAGCAGCTCCAGCCTACACCATGGGAATTAGCCATGGTCC
JSO-1966: pKD3 *cmR* amplification	GCAGAAAAATCAGCGAATAAATAGGTGTAGGCTGGAGCTGCTTC
JWO-1116: *Enterococcus faecium* E1007 phage phi1	GATTTGGCTGGTACGGAGTTGC
JWO-1117: *Enterococcus faecium* E1007 phage phi1	AGTTGCAGAGGAAAATGGGGG
JWO-1120: *Enterococcus faecium* E1007	GACCAGTTGCAAGGCGAAGG
JWO-1121: *Enterococcus faecium* E1007	TCACCGTTTGTCGAATCGCC
JSO-1745: *cmR* for pEVS143-empty (*kanR-cmR*); ligated with primer product used for pEVS143-empty (*kanR*)	GGCATTTATTCTCAGGATAATTGTTTCAGC
JWO-1046: GC-rich probe	GCCGCATGCCGCATGCCGCATGCCGCATGCCGCATGCCGCATGCCGCATGCCGCATGCCGCAT
JWO-1047: GC-rich complement	ATGCGGCATGCGGCATGCGGCATGCGGCATGCGGCATGCGGCATGCGGCATGCGGCATGCGGC
JWO-1044: AT-rich probe	CACACATTTGCAAATTTCGCAAATTTGCACAGATCTTGCAAATTTGCAAATTTGCAAATTTCA
JWO-1045: AT-rich complement	TGAAATTTGCAAATTTGCAAATTTGCAAGATCTGTGCAAATTTGCGAAATTTGCAAATGTGTG

Supplementary References

1. Huo, L. *et al.* Heterologous expression of bacterial natural product biosynthetic pathways. *Nat. Prod. Rep.* **36**, 1412–1436 (2019).
2. Arthur, J. C. *et al.* Intestinal Inflammation Targets Cancer-Inducing Activity of the Microbiota. *Science* **338**, 120–123 (2012).
3. Yang, Y., Gharaibeh, R. Z., Newsome, R. C. & Jobin, C. Amending microbiota by targeting intestinal inflammation with TNF blockade attenuates development of colorectal cancer. *Nat. Cancer* **1**, 723–734 (2020).
4. Bossuet-Greif, N. *et al.* The Colibactin Genotoxin Generates DNA Interstrand Cross-Links in Infected Cells. *mBio* **9**, (2018).
5. Dziubańska-Kusibab, P. J. *et al.* Colibactin DNA-damage signature indicates mutational impact in colorectal cancer. *Nat. Med.* **26**, 1063–1069 (2020).
6. Pleguezuelos-Manzano, C. *et al.* Mutational signature in colorectal cancer caused by genotoxic pks + E. coli. *Nature* **580**, 269–273 (2020).
7. Arndt, D. *et al.* PHASTER: a better, faster version of the PHAST phage search tool. *Nucleic Acids Res.* **44**, W16–W21 (2016).
8. Faïs, T. *et al.* Antibiotic Activity of Escherichia coli against Multiresistant Staphylococcus aureus. *Antimicrob. Agents Chemother.* **60**, 6986–6988 (2016).
9. Zhang, X. *et al.* Quinolone Antibiotics Induce Shiga Toxin-Encoding Bacteriophages, Toxin Production, and Death in Mice. *J. Infect. Dis.* **181**, 664–670 (2000).
10. Hendrix, R. W., Smith, M. C. M., Burns, R. N., Ford, M. E. & Hatfull, G. F. Evolutionary relationships among diverse bacteriophages and prophages: All the world's a phage. *Proc. Natl. Acad. Sci.* **96**, 2192–2197 (1999).
11. Mushegian, A. R. Are There 1031 Virus Particles on Earth, or More, or Fewer? *J. Bacteriol.* **202**, (2020).
12. Duerkop, B. A., Clements, C. V., Rollins, D., Rodrigues, J. L. M. & Hooper, L. V. A composite bacteriophage alters colonization by an intestinal commensal bacterium. *Proc. Natl. Acad. Sci.* **109**, 17621–17626 (2012).

13. Baba, T. *et al.* Construction of Escherichia coli K-12 in-frame, single-gene knockout mutants: the Keio collection. *Mol. Syst. Biol.* **2**, 2006.0008 (2006).
14. Eames, M. & Kortemme, T. Cost-Benefit Tradeoffs in Engineered lac Operons. *Science* **336**, 911–915 (2012).
15. Tomkovich, S. *et al.* Locoregional Effects of Microbiota in a Preclinical Model of Colon Carcinogenesis. *Cancer Res.* **77**, 2620–2632 (2017).
16. Owen, S. V. *et al.* Characterization of the Prophage Repertoire of African Salmonella Typhimurium ST313 Reveals High Levels of Spontaneous Induction of Novel Phage BTP1. *Front. Microbiol.* **8**, (2017).
17. Owen, S. V. *et al.* Prophages encode phage-defense systems with cognate self-immunity. *Cell Host Microbe* **29**, 1620-1633.e8 (2021).
18. Mallick, E. M. *et al.* A novel murine infection model for Shiga toxin–producing *Escherichia coli*. *J. Clin. Invest.* **122**, 4012–4024 (2012).
19. Novick, R. Properties of a cryptic high-frequency transducing phage in Staphylococcus aureus. *Virology* **33**, 155–166 (1967).
20. Úbeda, C., Barry, P., Penadés, J. R. & Novick, R. P. A pathogenicity island replicon in Staphylococcus aureus replicates as an unstable plasmid. *Proc. Natl. Acad. Sci.* **104**, 14182–14188 (2007).
21. Lebreton, F. *et al.* Tracing the Enterococci from Paleozoic Origins to the Hospital. *Cell* **169**, 849-861.e13 (2017).
22. Lindsey, R. L., Rowe, L. A., Batra, D., Smith, P. & Strockbine, N. A. PacBio Genome Sequences of Eight Escherichia albertii Strains Isolated from Humans in the United States. *Microbiol. Resour. Announc.* **8**, e01663-18.
