# Supplementary material for: The bacterial toxin colibactin triggers prophage induction
Source: Nature. 2022 Feb 23;603(7900):315–20. doi: 10.1038/s41586-022-04444-3 (PMC8907063; doi:10.1038/s41586-022-04444-3)
Supplement: Supplementary file 2 — Reporting Summary [file 41586_2022_4444_MOESM2_ESM.pdf]

nature research

Corresponding author(s): Emily P. Balskus

Last updated by author(s): January 14, 2022

Reporting Summary

Nature Research wishes to improve the reproducibility of the work that we publish. This form provides structure for consistency and transparency in reporting. For further information on Nature Research policies, see our Editorial Policies and the Editorial Policy Checklist.

Statistics

For all statistical analyses, confirm that the following items are present in the figure legend, table legend, main text, or Methods section.

n/a	Confirmed	
☐	☒	The exact sample size (*n*) for each experimental group/condition, given as a discrete number and unit of measurement
☐	☒	A statement on whether measurements were taken from distinct samples or whether the same sample was measured repeatedly
☒	☐	The statistical test(s) used AND whether they are one- or two-sided<br>*Only common tests should be described solely by name; describe more complex techniques in the Methods section.*
☒	☐	A description of all covariates tested
☐	☒	A description of any assumptions or corrections, such as tests of normality and adjustment for multiple comparisons
☐	☒	A full description of the statistical parameters including central tendency (e.g. means) or other basic estimates (e.g. regression coefficient) AND variation (e.g. standard deviation) or associated estimates of uncertainty (e.g. confidence intervals)
☒	☐	For null hypothesis testing, the test statistic (e.g. *F*, *t*, *r*) with confidence intervals, effect sizes, degrees of freedom and *P* value noted<br>*Give P values as exact values whenever suitable.*
☒	☐	For Bayesian analysis, information on the choice of priors and Markov chain Monte Carlo settings
☒	☐	For hierarchical and complex designs, identification of the appropriate level for tests and full reporting of outcomes
☒	☐	Estimates of effect sizes (e.g. Cohen's *d*, Pearson's *r*), indicating how they were calculated

Our web collection on statistics for biologists contains articles on many of the points above.

Software and code

Policy information about availability of computer code

Data collection	Gen5 v3 for collection of of growth- and reporter-based experiments and Bio-Rad CFX Manager 3.0 for collection of qPCR based experiments.
Data analysis	Software used to analyze the data generated in this study consisted of: GraphPad Prism 9 for analysis of growth- and reporter-based experiments; Bio-Rad CFX Manager 3.0 for quantification and analysis of qPCR data; ImageJ 1.53c for colony counting in competition experiments; and Geneious Prime 2020 for analysis of publicly available data and primer design. Data are presented as the mean ± std unless otherwise indicated in the figure legends. The number of independent biological replicates for each experiment is indicated for each experiment and included in the legend.

For manuscripts utilizing custom algorithms or software that are central to the research but not yet described in published literature, software must be made available to editors and reviewers. We strongly encourage code deposition in a community repository (e.g. GitHub). See the Nature Research guidelines for submitting code & software for further information.

Data

Policy information about availability of data

All manuscripts must include a data availability statement. This statement should provide the following information, where applicable:

- Accession codes, unique identifiers, or web links for publicly available datasets
- A list of figures that have associated raw data
- A description of any restrictions on data availability

All unprocessed plaque assay images (Figures 1c, 2a, 2b, 3a and Extended Data Figures 1c, 2c, 2f, 2h, 3g) and source data (Figures 1b, 1d, 2c, 2d, 2f, 2g, 2h, 3a, 3c, and Extended Data Figures 1a, 1b, 1d, 1e, 2b, 2e, 2g, 3a, 3d, 3e, 3f, 4b, 4c) generated in the course of this study are available without restriction and deposited on Zenodo (doi: 10.5281/zenodo.4683078). Total PFU, and CFU and growth data (Supplemental Discussion Figure 1) are available in Supplementary Tables 5 and 6, respectively. Identifiers for all entries in NCBI BLAST results are listed in Supplementary Table 1. Protein accession numbers for the relevant ClbS sequences tested in

this study are as follows: E. coli CFT073 (WP_000290498), M. theicola (PNS10644), S. erythrinae (WP_132453050), G. quercinecans (WP_095844971), S alvi (WP_025331471), F. perrara (WP_039103908), Metakosakonia sp./K. intestini (BBE76153/ PWF54517), E. albertii (WP_000115842), E. coli 69 (QDM73539), D. dadantii (WP_038909824), B. longum (WP_193641739) . Accession numbers used for the design of qPCR primers and reporter construction are: E. faecium E1007 (AHWP00000000), E. coli BW25113/lambda (NZ_CP009273/NC_001416.1), E. albertii 07-3866 (NZ_CP030781), and Metakosakonia sp. (AP018756). Accession and identifier information can be found at NCBI.

Field-specific reporting

Please select the one below that is the best fit for your research. If you are not sure, read the appropriate sections before making your selection.

☒ Life sciences ☐ Behavioural & social sciences ☐ Ecological, evolutionary & environmental sciences

For a reference copy of the document with all sections, see nature.com/documents/nr-reporting-summary-flat.pdf

Life sciences study design

All studies must disclose on these points even when the disclosure is negative.

Sample size	Sample size was chosen as three biological replicates, matching the standard in the microbiology field [e.g., Erez and Steinberger-Levy et al. Nature 541, 488–493 (2017)]. All datapoints displayed in this study are available in the source data for others to access and analyze. Means and standard deviations are plotted; no additional statistical analyses were performed [see D.L. Vaux "Know when your numbers are significant. Nature 492, 180–181 (2012)].
Data exclusions	No data were excluded.
Replication	At least three replicates were used for each experiment. All data points were plotted and are available in the source data file. No data were excluded, and all replicates were therefore considered "successful" measurements.
Randomization	Randomization was not formally implemented in this study, however, the choice of wells and positioning of culture tubes used in any given experiment was not pre-assigned and was therefore chosen randomly at the time of setup.
Blinding	Blinding was not formally applied in this study. The investigators setting up the assays also analyzed the data. The strains used in each experiment were assigned numbers that were cross-checked with the corresponding sample names/treatments only at the time the data were plotted.

Reporting for specific materials, systems and methods

We require information from authors about some types of materials, experimental systems and methods used in many studies. Here, indicate whether each material, system or method listed is relevant to your study. If you are not sure if a list item applies to your research, read the appropriate section before selecting a response.

Materials & experimental systems

n/a	Involved in the study	
☒	☐	Antibodies
☒	☐	Eukaryotic cell lines
☒	☐	Palaeontology and archaeology
☐	☒	Animals and other organisms
☒	☐	Human research participants
☒	☐	Clinical data
☒	☐	Dual use research of concern

Methods

n/a	Involved in the study	
☒	☐	ChIP-seq
☒	☐	Flow cytometry
☒	☐	MRI-based neuroimaging

Animals and other organisms

Policy information about studies involving animals; ARRIVE guidelines recommended for reporting animal research

Laboratory animals	No laboratory animals were directly used in this study.
Wild animals	No wild animals were used in this study.
Field-collected samples	No new field-collected samples were collected in this study. Bacteriophages and bacteria were used from previously published sources, listed in the strain table.
Ethics oversight	No ethical approval or guidance was required for this study.

Note that full information on the approval of the study protocol must also be provided in the manuscript.
